# Nephrotoxicity and kidney outcomes in pediatric oncology patients

**DOI:** 10.1093/ndt/gfaf169

**Published:** 2025-08-22

**Authors:** Paulien A M A Raymakers-Janssen, Nils Leitzinger, Gerrit van den Berg, Joppe Nijman, Mieke I Triest, Ellen Kilsdonk, Inge A van Kessel, Lidwien M Hanff, Martine van Grotel, Marc H W A Wijnen, Roelie M Wösten-van Asperen, Marc R Lilien, Marry M van den Heuvel-Eibrink, Marta Fiocco

**Affiliations:** Department of Pediatric Intensive Care, Wilhelmina Children's Hospital/ University Medical Center Utrecht, Utrecht, The Netherlands; Princess Máxima Center for Pediatric Oncology, Utrecht, The Netherlands; Mathematical Institute, Leiden University, Leiden, The Netherlands; Department of Pediatric Nephrology, Wilhelmina Children's Hospital/University Medical Center Utrecht, Utrecht, The Netherlands; Department of Pediatric Intensive Care, Wilhelmina Children's Hospital/ University Medical Center Utrecht, Utrecht, The Netherlands; Princess Máxima Center for Pediatric Oncology, Utrecht, The Netherlands; Princess Máxima Center for Pediatric Oncology, Utrecht, The Netherlands; Department of Pediatric Intensive Care, Wilhelmina Children's Hospital/ University Medical Center Utrecht, Utrecht, The Netherlands; Princess Máxima Center for Pediatric Oncology, Utrecht, The Netherlands; Princess Máxima Center for Pediatric Oncology, Utrecht, The Netherlands; Princess Máxima Center for Pediatric Oncology, Utrecht, The Netherlands; Department of Pediatric Intensive Care, Wilhelmina Children's Hospital/ University Medical Center Utrecht, Utrecht, The Netherlands; Department of Pediatric Nephrology, Wilhelmina Children's Hospital/University Medical Center Utrecht, Utrecht, The Netherlands; Princess Máxima Center for Pediatric Oncology, Utrecht, The Netherlands; Division of Child Health, Wilhelmina Children's Hospital/University Medical Center Utrecht, Utrecht, The Netherlands; Princess Máxima Center for Pediatric Oncology, Utrecht, The Netherlands; Mathematical Institute, Leiden University, Leiden, The Netherlands; Department of Medical Statistics and Bioinformatics, Leiden University Medical Center, Leiden, The Netherlands

**Keywords:** AKI, CKD, epidemiology, nephrotoxicity, pediatric cancer patients

## Abstract

**Background:**

Acute kidney injury (AKI) is a serious complication during pediatric cancer treatment. Nephrotoxic medication may increase the risk of developing AKI, which may necessitate modifications to standard treatment and may also increase the risk of chronic kidney disease (CKD). This study investigates the incidence of AKI, the impact of nephrotoxic medications and the association between AKI and the development of CKD.

**Methods:**

In this retrospective national cohort study, we analyzed 1525 pediatric cancer patients treated at the Princess Máxima Center between 2015 and 2021. AKI was classified using KDIGO criteria based on serum creatinine. The effect of nephrotoxic medications and other risk factors on AKI incidence and progression was assessed by using a cause specific hazard regression model. The cumulative incidence of AKI was estimated with a competing risk model with death as competing event. The effect of risk factors on CKD, defined as an eGFR <90 ml/min/1.73 m² 1 year after cancer treatment, was evaluated with a logistic regression.

**Results:**

We included 1525 patients, 37% experienced AKI. A competing risk model identified treatment with ifosfamide, amphotericin B, acyclovir, and busulfan as strong, independent risk factors for a first episode of AKI. Older age was also associated with an increased risk of AKI.

At 1-year follow-up (*n* = 1159), 13.6% had CKD (eGFR <90 ml/min/1.73 m²), and 2.8% had an eGFR <60. AKI (occurred during treatment) was the strongest predictor of CKD: a single AKI episode increased the risk 2.6-fold, while more episodes increased it nearly 16-fold. Nephrectomy was also identified as independent risk factors for CKD.

**Conclusion:**

Acute kidney injury (AKI) is common in children with cancer and is strongly associated with an increased risk of chronic kidney disease (CKD). Awareness is crucial for high-risk patients, particularly those receiving nephrotoxic medications, with a history of multiple AKI episodes or a prior nephrectomy. Comprehensive monitoring strategies should be implemented at diagnosis, during therapy, and during the post-treatment period to enable early detection and timely intervention, ultimately reducing the risk of AKI and its progression to CKD.

KEY LEARNING POINTS **What was known:**

- AKI is a complication of pediatric cancer treatment.

**This study adds:**

- Acute kidney injury (AKI) is common in children with cancer and is strongly associated with an increased risk of chronic kidney disease (CKD).
- This study investigates also the impact of pediatric cancer medication to the occurrence of AKI.

**Potential impact:**

- Monitoring AKI during treatment is essential in patients at risk.
- In addition, kidney function should be monitored in pediatric cancer survivors.
- Reducing the occurrence of AKI during treatment may decrease the risk of CKD development in pediatric cancer survivors.

## INTRODUCTION

Pediatric cancer patients suffer from several adverse events during treatment. Acute kidney injury (AKI) is a serious, but often disguised complication with a multifactorial etiology [1–3]. Factors that potentially determine the occurrence of AKI in pediatric cancer patients are anatomical obstruction, tumor lysis syndrome, treatment with cancer and supportive care medication (e.g. chemotherapy, antifungal agents, antibiotics), surgery (nephrectomy), radiotherapy, and sepsis leading to multi organ failure [1, 2, 4–7].

The occurrence of AKI has been reported to be associated with increased duration of hospital stay, due to accompanying fluid overload, hypertension, and electrolyte disturbances requiring hospitalization, and to often lead to (temporary) amendment of regular cancer treatment (dose adjustments or treatment delay) [2, 4].

There are only a few studies exploring potential risk factors associated with the occurrence of AKI episodes in pediatric patients. Moffett and colleagues identified administered medication such as chemotherapeutics, antibiotics such as piperacillin, as well nephrectomy as the major risk factor for AKI [8]. However, this study focused on potential nephrotoxic medication in the general pediatric population.

In the adult cancer population, several studies reported the incidence of AKI during cancer treatment, varying from 12% to 25.8% [3, 7, 9, 10]. Also, it was shown that some patients developed chronic kidney disease (CKD) after experiencing a period of AKI [2, 11].

Studies on AKI in children during cancer treatment and the development toward CKD are scarce [11–14]. Park *et al.* were the first to discuss the occurrence of AKI within the first year after cancer diagnosis in a single center cohort of 1868 pediatric cancer patients and reported an AKI incidence of 52.6% [2].

Furthermore, the association between the occurrence and duration of AKI and the development of CKD has not been explored comprehensively in full cohorts of pediatric cancer patients. Given the lack of available full cohort studies that explored relevant factors associated with the development of AKI and subsequent CKD in pediatric cancer patients, we performed a national cohort study in pediatric cancer patients.

In this study we estimated the incidence of AKI in our national cohort of pediatric cancer patients during treatment. The effect of individual medications on the occurrence of AKI episodes was also investigated. Finally, the impact of AKI episode(s) on the development of CKD 1 year after cancer treatment ended was studied.

## MATERIALS AND METHODS

### Study design

All pediatric cancer patients (identified using the Máxima data warehouse), who started and completed treatment of cancer between 1 January 2015 and 1 January 2021, in the Princess Máxima Center for Pediatric Oncology, Utrecht, the Netherlands were included. Data collection from the electronic medical records (EMRs) was performed according to GDPR procedures, approved by the Biobanking and Data Access use procedure of the Princess Máxima Center (PMCLAB2019.065). The study was submitted to the institutional ethical review board (reference / METC number 19–449/C). Need for additional informed consent was waived.

**Figure 1:**
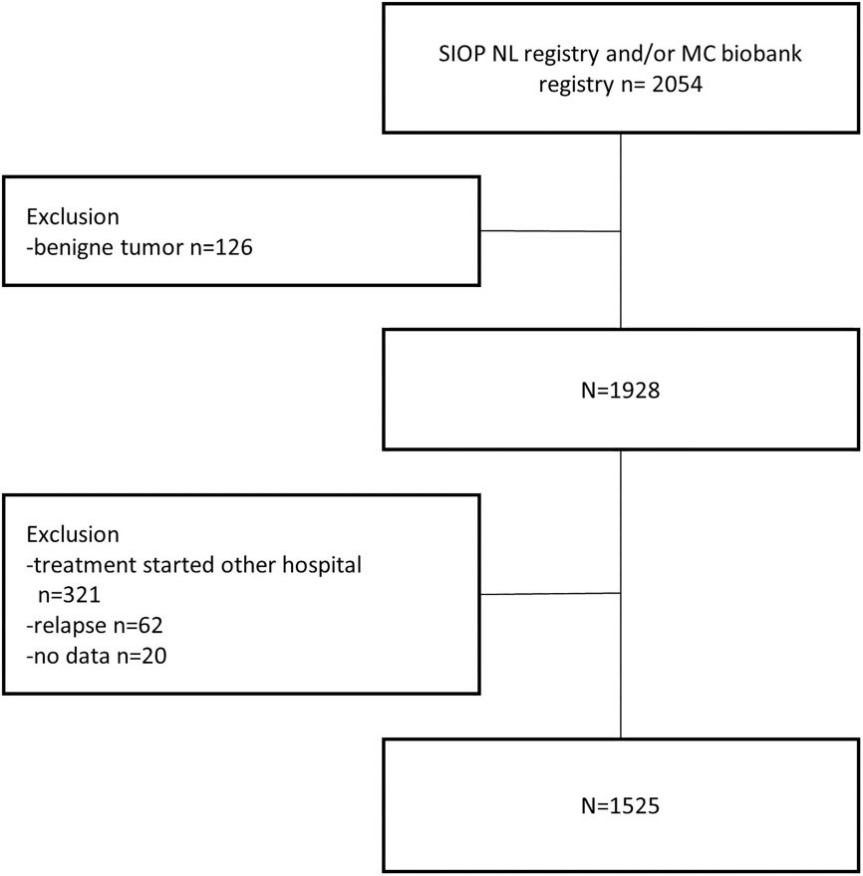
Flow diagram for the selection of the eligible study population.

Eligible patients were younger than 19 years at cancer diagnosis. Patients who had a first episode of AKI before starting the cancer treatment, including those who received renal replacement therapy (RRT) before start of the study, were excluded. Other exclusion reasons were benign tumors (*n* = 126), treatment (started) in other hospital (*n* = 321), relapse treatment (*n* = 62), and no available data (*n* = 20) (see Fig. 1 and Supplementary S1 for additional details).

### Data collection and measurements

Anonymized data were extracted from the EMRs, including demographics, type of cancer diagnosis, disease stage, details about cancer treatment, and supportive care (including chemotherapy, supportive care co-medication, radiotherapy, and surgery).

Renal function data included serum creatinine (sCr) levels and estimated glomerular filtration rate (eGFR). Urine albumin/creatinine ratio was not available in most children (see Supplementary S2).

AKI was defined and classified according to the Kidney Disease Improving Global Outcomes (KDIGO) criteria [12], which are based on changes of serum creatinine as well as urine production [12] (see Supplementary S3). However, urine output criteria were not used for the assessment as nephrotoxic AKI is usually non-oliguric in nature [13] and urine output was not precisely documented in the EMRs. Each serum Cr level was compared to the baseline defined as the lowest serum Cr in the previous seven days. If no serum Cr levels were available, the lowest level in the 3 months preceding hospital admission was used as baseline. If a patient displayed the same creatinine levels consistently for seven consecutive days, this value was considered the new baseline for that patient. We categorized the duration of AKI episodes as transient AKI (<48 h), persistent AKI (>48 h), and AKD (acute kidney disease, >7 days) [14]. A minimum of 48 h is necessary to separate two distinct AKI episodes [14].

In this study the presence of CKD 1 year after completion of cancer treatment was defined as a low eGFR (<90 ml/min/1.73 m^2^) for >3 months and/or proteinuria (protein-to-creatinine ratio >20 mg/mmol at age ≥2 yr and >50 mg/mmol at age <2 yr). Due to the lack of measurements of proteinuria attributed to the retrospective nature of the study, we classified CKD only based on eGFR. A final eGFR of <90 ml/min/1.73 m^2^ was defined as impaired renal function after the end of therapy. The eGFR was calculated using the modified Schwartz formula (see Supplementary 3 for more details).

### Selection of anticipated nephrotoxic medication for the current study

Information about nephrotoxic medications was extracted from the central electronic pharmacy environment of the medical records during the study period. All prescribed medications during the study period were reviewed by the research team for previously described evidence of nephrotoxicity. Medication is classified as nephrotoxic if this was described as nephrotoxic in the pharmaceutic formulary of the Dutch hospitals, classified in one of four categories (>10%, 1–10%, 0.1–1%, or <0.1%). We also classified medication as nephrotoxic if a pharmacologist, pediatric oncologist, nephrologist, or renal nurse reviewed it as nephrotoxic. Among 1200 different prescribed drugs (many of which were the same medication but under different brands) in our hospital, the study team identified 51 medications as potentially nephrotoxic (see Supplementary Table S1 for details). Nephrotoxic medication exposure was defined as administration within the 14 days preceding the AKI event, based on clinical considerations regarding the expected time frame for nephrotoxic injury. Only exposures within this window were included in the analyses. We excluded medication that was administered on fewer than 10 occasions in the entire study population.

### Statistical analyses

Categorical data are presented as frequencies and percentages. For continuous data summary statistics of mean, standard deviation, median, and interquartile range are shown.

**Figure 2:**
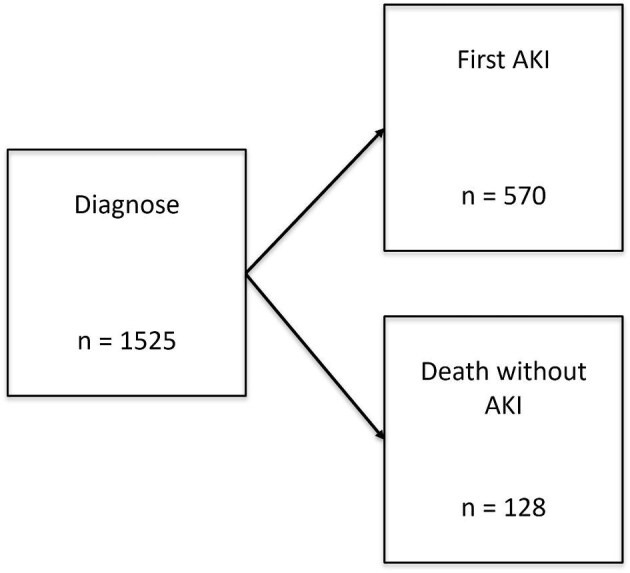
Competing risk model with two events: AKI first episode and death.

A competing risk model (see Fig. 2) [15] considering AKI during cancer treatment as the event of interest and death as competing event, was employed to estimate the cumulative incidence of a first AKI episode from the time from diagnosis. Gray's test [16] was used to assess differences in cumulative incidence for different nephrotoxic medication. To study the impact of nephrotoxic medication on the first AKI episode, a cause specific hazard regression model was estimated. Cause specific hazard ratio (HR_CS_), along with 95% confidence interval (CI) are reported. To study the association between AKI during treatment and the development of CKD 1 year after treatment completion, a logistic regression model was estimated. Only patients with at least 1 year of follow-up were included in the analyses. Well known risk factors for CKD such as age, gender, nephrectomy, and radiotherapy were also included in the model [2, 17–20, 21]. Odds ratio (OR) with 95% confidence intervals are reported.

Statistical analyses were performed in IBM SPSS Statistics for Windows, version 29 (IBM corp., Armonk, NY, USA). All analyses concerning the competing risk model were performed in the R software environment [22] with the library mstate [23].

## RESULTS

**Figure 3:**
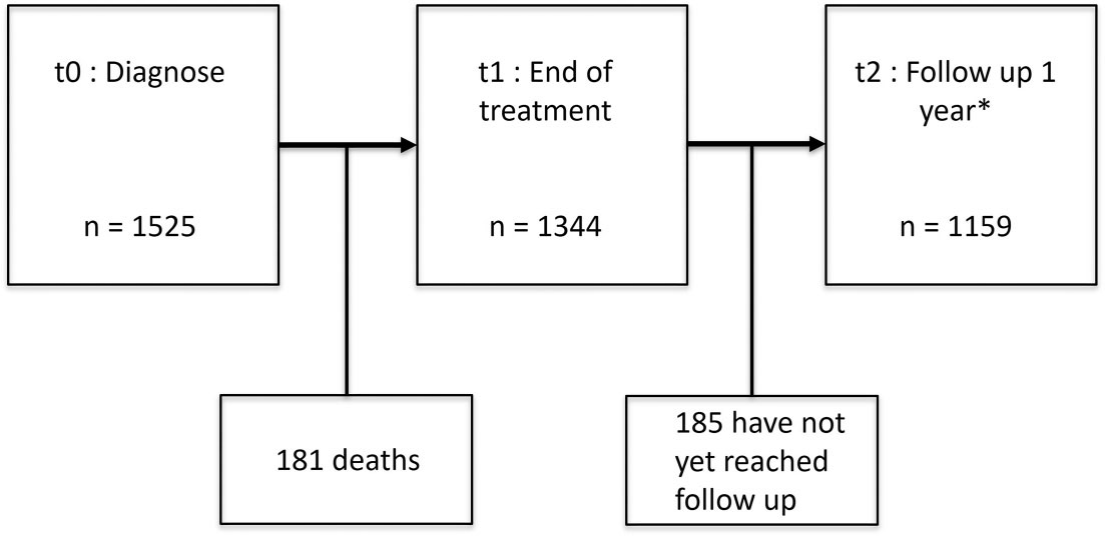
Cohort during the study period.

A total of 2054 patients were diagnosed with cancer during the study period: 1525 (845 male, 680 female) were eligible for analysis (Fig. 1). The median age of the patients at inclusion of the study was 5.9 years [interquartile range (IQR) 2.3–13.3]. Renal tumors, neuroblastoma, brain tumors, soft tissue tumors, and acute leukemia were the most common cancer types in our study population (Table 1). Follow-up data at 1 year after completion of cancer therapy were available in 1159 patients; 185 patients had not yet reached 1 year post-treatment at the time of data collection and in total 181 patients (11.8%) died during the study period, 128 patients died without experiencing an AKI episode (Figs. 2 and 3).

**Table 1:** Patient characteristics.

Variables	All patients *N* = 1525	AKI *N* = 570	No AKI *N* = 937
Age, yrs, median (IQR)	5.9 (2.3–13.3)	5.7 (2.7–13.2)	6.2 (2.2–13.3)
Sexe, male, *n* (%)	845 (55)	323 (38)	522 (62)
Oncological diagnosis			
ALL	307	162	145
AML	62	40	22
MDS/JMML	5	5	0
Renal	147	104	43
Adrenal	151	80	68
NHL	80	42	38
HL	121	25	96
Brain/ CNS	192	25	167
Soft tissue	118	29	89
Bone	101	31	70
Liver	29	13	16
Germcell	51	6	45
LCH	39	5	34
other	122	16	106
IC admission, *n* (%)	418 (27)	244/418 (58)	174/418 (42)
Nephrectomy, *n* (%)	163 (11)	110/163 (67)	53/163 (33)
CRRT, *n* (%)	31 (2)	31/31 (100)	
HSCT, *n* (%)	156 (10)	86/156 (55)	70/156 (45)
Death, *n* (%)	181 (12)	53/181 (29)	128/181 (71)

ALL, acute lymphoblastic leukemia; AML, acute myeloid leukemia; MDS, myelodysplasia; JMML, juvenile myelomonocytic leukemia; NHL, non-Hodgkin lymphoma; HL, Hodgkin lymphoma; CNS, central nervous system; LCH, Langerhans cell histiocytosis; IC, intensive care; CRRT. continuous RRT; HSCT, hematopoietic stem cell transplantation.

### Incidence of acute kidney injury

Among the eligible patients, 570 (37.4%) experienced at least one episode of AKI during their treatment course (Table 2). Among the patients who developed AKI during treatment, 336 (59%) experienced one episode, 137 (24%) developed two episodes, while 97 (17%) developed three or more episodes (see Supplementary Fig. S1).

**Table 2:** Incidence of AKI during cancer treatment.

					AKI episodes *n* = 570		
Diagnosis	Cohort *n*	Death *n*	AKI *n*	First day of AKI days median (IQR)	1 episode of AKI	2 episodes of AKI	3 or more episodes of AKI
All tumors	1525	181	570	87.5 (38.0–161.2)	336	137	97
ALL	307	14	162	98 (54–174)	83	45	34
AML	62	9	40	77 (40–135)	12	13	15
MDS/JMML	5	0	5	159 (116–228)	2	1	2
Renal	147	6	104	42 (31–58)	85	13	6
Adrenal	151	43	80	140 (60–318)	44	20	16
NHL	80	4	42	69 (35–114)	26	11	5
HL	121	2	25	72 (39–96)	18	5	2
Brain/CNS	192	45	25	121 (64–290)	18	6	1
Soft tissue	118	23	29	189 (69–279)	20	4	5
Bone	101	22	31	134 (52–265)	14	12	5
Liver	29	6	13	36 (14–99)	6	5	2
Germcell	51	2	6	106 (33–148)	4	1	1
LCH	39	0	5	124 (45–249)	5	0	0
other	122	5	16	42 (15–119)	9	4	3

ALL, acute lymphoblastic leukemia; AML, acute myeloid leukemia; MDS, myelodysplasia; JMML, juvenile myelomonocytic leukemia; NHL, non-Hodgkin lymphoma; HL, Hodgkin lymphoma; CNS, central nervous system; LCH, Langerhans cell histiocytosis; First day of AKI, date of cancer diagnosis = day 0.

The highest incidence of AKI during treatment was observed in patients with AML (66%), NHL (53%), and kidney tumors (71%). In patients with ALL, adrenal tumors, NHL, and soft tissue tumors, >50% developed two or more episodes of AKI (Table 2).

During the first AKI episode, severity of AKI was classified as stage 1 (65%), stage 2 (24%) or stage 3 (11%) Among patients who experienced three or more AKI episodes, almost 70% were classified as AKI stage 2–3. The duration of AKI episode increased with the number of AKI episodes during treatment (see Supplementary Table S2 for more details).

### Impact of nephrotoxic medication on AKI

Age was associated with a higher risk of AKI (HR_CS_ 1.13; 95% CI 1.11–1.15). Carboplatin and diclofenac were associated with AKI (HR_CS_ 0.58; 95% CI 0.38–0.88 and HRcs 0.39; 95% CI 0.29–0.53, respectively). Ifosfamide (HRcs 2.99; 95% CI 2.30–3.88), busulfan (HRcs 1.53; 95% CI 1.03–2.25), acyclovir (HRcs 1.53; 95% CI 1.03–2.26), and amphotericin B (HR_CS_ 2.08; 95% CI 1.53–2.8) were associated with significantly increased hazard of developing AKI (see Table 3). Significant differences in the cumulative incidence of AKI were observed for several nephrotoxic medications, including ifosfamide, amphotericin B, busulfan, diclofenac, clofarabine (Evoltra), acyclovir, morphine, carboplatin, etoposide, caspofungin, and pentamidine.

**Table 3:** Multivariable cause specific hazard regression model for AKI first episode. Estimated cause specific hazard ratio (HR_CS_) and 95% CI. No medication is the reference category.

Risk factors	HR_CS_ (95%CI)
Age	1.13 (1.11–1.15)
Carboplatin	0.58 (0.38–0.88)
Ifosfamide	2.99 (2.30–3.88)
Busulfan	1.53 (1.03–2.25)
Acyclovir	1.53 (1.03–2.26)
Diclofenac	0.39 (0.29–0.53)
Amphotericin B	2.08 (1.53–2.81)

The cumulative incidence plots and Gray's test results suggest that patients receiving these agents experienced higher or more rapidly occurring AKI events compared to those not exposed, highlighting clinically relevant differences in AKI risk profiles.

These detailed incidence curves and test statistics are provided in Supplemental Fig. S2.

### Impact of AKI during treatment on the development of CKD 1 year after end of treatment

A total of 1159 patients had a follow-up of at least 1 year after completion of cancer therapy at time of analysis (see Fig. 3). One hundred and fifty-eight patients (13.6%) had an eGFR below 90 ml/min/1.73 m^2^ (CKD2), with a median of 71 ml/min/1.73 m² (IQR 64–77), and 33 (2.8%) below 60 ml/min/1.73 m^2^, with a median of 37 ml/min/1.73 m² (IQR 26–50) 1 year after completion of treatment. In multivariable logistic regression, age (OR 1.08, 95% CI 1.05–1.12), and nephrectomy (OR 3.53, 95% CI 1.90–6.59) were significantly associated with CKD at 1 year post-treatment. AKI during therapy was strongly associated with CKD: patients with one AKI episode had an increased risk (OR 2.60, 95% CI 1.62–4.15), and those with more than one had a markedly higher risk (OR 15.90, 95% CI 10.44–24.20), gender (OR 1.37, 95% CI 0.96–1.96, *P* = .086), and radiotherapy (OR 1.21, 95% CI 0.47–3.12, *P* = .693) were not significantly associated with CKD (see Table 4). CKD outcomes stratified by duration of AKI are listed in Supplementary Table S3.

**Table 4:** Multivariable logistic regression model for CKD. OR along with 95% CI are reported.

Variable	OR (95% CI)
Sex, boy	1.37 (0.96–1.96)
Age, y	1.08 (1.05–1.12)
Nephrectomy	3.53 (1.90–6.59)
Radiotherapy	
Yes	1.21 (0.47–3.12)
AKI during treatment	
No AKI (ref.)	Ref
1 episode of AKI	2.60 (1.62–4.15)
>1 episode of AKI	15.90 (10.44–24.20)

## DISCUSSION

This study investigated the impact of nephrotoxic medications on the risk of developing AKI during pediatric cancer treatment, using a competing risk model. In addition, the impact of AKI during treatment on the development of CKD after 1 year from the end of treatment was subject of our study.

Approximately one-third of pediatric cancer patients in our cohort experienced at least one AKI episode during treatment; 41% developed more than one episode and 17% three or more. Two previous studies investigated the occurrence of AKI in hospitalized pediatric cancer patients [2, 24]. Xiong and colleagues reported in a multicenter study in China an AKI incidence of 16.9% (1657/9825) [24]; this is lower compared to the incidence in our cohort, which might be because only the first hospitalization had been considered in that study. In a single center study in South Korea, an incidence of 52.6% (983/1868) was found [2]. Studies that investigated the occurrence of AKI in specific subsets of pediatric oncology patients, reported prevalence ranges from 10% to 74% [24–27].

Our study identified ifosfamide, amphotericin B, busulfan, and acyclovir as being significantly associated with the occurrence of a first AKI episode during pediatric cancer treatment. These associations confirm findings of previous studies that highlighted the nephrotoxic potential of these agents.

Ifosfamide is well known for its nephrotoxic effects in children, particularly causing proximal tubular dysfunction and a Fanconi-like syndrome. The mechanism of toxicity is complex, but involves mitochondrial damage and increased oxidative stress [28]. Up to 30% of children receiving ifosfamide-based chemotherapy develop nephrotoxicity, especially those who receive high cumulative doses or in combination with other nephrotoxic drugs [29–32]

Conventional amphotericin B, is known to induce renal vasoconstriction and direct tubular toxicity, resulting in electrolyte disturbances and reduced glomerular filtration rate. High incidences of AKI have been reported in pediatric oncology and transplant patients treated with this drug [33]. Busulfan, mainly used as conditioning agent prior to hematopoietic stem cell transplantation, has also been shown to be associated with AKI. While data in pediatric populations are limited, recent studies suggest that busulfan may induce tubular injury and contribute to AKI, especially in combination with other chemotherapeutic agents [34]. Acyclovir, which is known to potentially cause crystal-induced nephropathy, particularly when administered intravenously at high dosages or with poor hydration. Retrospective pediatric studies reported AKI rates up to 7%, and recommend close monitoring of renal function during treatment [35]. These findings support careful use and monitoring of toxicity of these agents, particularly in patients with prior AKI.

Our cumulative incidence analyses further illustrate the clinical relevance of these nephrotoxic agents. Using Gray's test for competing risks, we found that patients exposed to ifosfamide and amphotericin B had a significantly higher and faster cumulative incidence of AKI compared to non-users. This means that not only do these medications increase the overall risk of AKI, but they also appear to accelerate its onset during treatment.

These findings highlight that early kidney damage may develop rapidly following exposure, especially when these drugs are used in combination with other nephrotoxic agents or in patients with pre-existing risk factors. This underscores the need for careful risk–benefit assessment, individualized dosing regimens, and intensified monitoring of renal function from the start of treatment.

Furthermore, this study investigates the effect of the occurrence and duration of AKI episodes during treatment on the development of CKD within 1 year post-cancer treatment. In our national cohort, older age and nephrectomy were significantly associated with CKD at 1 year post-treatment. This study shows the existing knowledge that AKI during pediatric cancer treatment is a strong independent risk factor for CKD 1 year after completion of therapy.

Patients with one AKI episode had a 2.6-fold increased risk, and those with more than one episode had nearly a 16-fold increased risk of developing CKD. These findings provide robust evidence in support of the hypothesis that recurrent AKI episodes contribute to long-term kidney damage, even in pediatric patients. Several previous late effect studies have identified similar risk factors for CKD, including nephrectomy, ifosfamide, cisplatin, gentamycin, and radiotherapy [17, 21, 36]. Only a meta-analysis in adults, by Coca *et al.*, has shown an association between AKI and the subsequent development of CKD [pooled OR of 8.8 (95% CI 3.1–25.5)] an association that our study now confirms in the pediatric population. That study highlighted the need for further interpretation of underlying pathophysiological mechanism [37].

In this study, 13% of young patients had an eGFR <90 ml/min/1.73 m^2^, while in 3% it was decreased below 60 ml/min/1.73 m^2^ at 1 year after completion of cancer treatment. These findings underscore the importance of closely monitoring patients with AKI episodes, those receiving nephrotoxic medications, those undergoing nephrectomy during cancer therapy, and also those after the end of treatment.

This study has some limitations. We focused on nephrotoxic medications as predictors of AKI. Although these agents are well-recognized risk factors, patient- and disease-specific variables (e.g. tumor type, HSCT, ICU admission, nephrectomy, comorbidities, and illness severity) may also contribute to AKI risk.

Given the moderate number of AKI events and the large number of potential covariates, we deliberately avoided an overparameterized model that could compromise statistical validity and interpretability.

We did not include cumulative medication dosages in the analysis due to the unavailability of reliable total cumulative dose at the time of study initiation. Future studies should incorporate clinical variables in the model. Second, as in almost all pediatric studies, sCr was adopted as an indicator of AKI despite its limitation in pediatric cancer patients because of effects of nutritional and fluid status (dilutional effect of fluid overload) on sCr levels. Furthermore, sCr can remain unchanged until more than half of the nephrons have lost their function. This suggests that the reported incidence of AKI, may be underestimated. Future studies should consider alternative biomarkers such as Cystatin C and NGAL for more accurate AKI detection in this specific patient population [38–40].

Our results may have important implications for daily care. Implementation of an automatized alert system in electronic healthcare system might help with early AKI detection, enabling early measures to prevent irreversible kidney damage [39]. Recently, Goldstein *et al.* developed a systematic electronic medical record screening with a decision support process and showed the efficacy of such systems in reducing AKI rates from 1.7 to 1.3 episodes per 1000 patient days. They showed that part of the nephrotoxic medication burden may be avoidable and that medication-related AKI should be considered as a potentially modifiable adverse event [41]. However, there is no treatment for AKI other than supportive care. Hence, reducing exposure to nephrotoxic agents remains a cornerstone of primary AKI prevention.

Thereby, by refining our understanding of medication-associated AKI risks and implementing early detection strategies, in the future clinicians may be able to optimize patient care outcomes during pediatric cancer treatment.

## CONCLUSION

Our national cohort study, shows a high prevalence (one-third) of at least one AKI episode in pediatric cancer patients during treatment. We showed that especially ifosfamide and amphotericin B induce AKI very early during treatment. The occurrence of AKI during treatment, nephrectomy, and an older age at diagnosis was strongly associated with the development of CKD 1 year post-therapy: patients with one AKI episode had a 2.6-fold increased risk, while those with multiple AKI episodes had a nearly 16-fold increased risk of CKD. Therefore, awareness is important and monitoring of renal function at diagnosis, during treatment, and after treatment is strongly recommended to prevent AKI and CKD as much as possible. In addition, development of intervention strategies to avoid early and long-term kidney injury is warranted in children with cancer.

## Supplementary Material

gfaf169_Supplemental_File

## Data Availability

The data supporting the findings of this study are not publicly available due to participant confidentiality. For the researchers and the analysis, all data have been anonymized or pseudonymized to ensure that individuals cannot be identified.
