# Supplementary material for: Nephrotoxicity and kidney outcomes in pediatric oncology patients
Source: Nephrol Dial Transplant. 2025 Aug 22;41(2):345–52. doi: 10.1093/ndt/gfaf169 (PMC12855606; doi:10.1093/ndt/gfaf169)
Supplement: gfaf169_Supplemental_File [file gfaf169_supplemental_file.docx]

**Electronic Supplementary Material**

**Nephrotoxicity and kidney outcomes in a national cohort of pediatric oncology: insights into Acute Kidney Injury and progression to Chronic Kideney Disease**

Paulien AMA Raymakers-Janssen^1,2^, Nils Leitzinger^3^, Gerrit van de Berg5, Joppe Nijman^1^ ,Mieke I Triest^2^, Ellen Kilsdonk^2^ , Inge A. van Kessel^1^, Lidwien M Hanff^2^, Martine van Grotel^2^, Marc HWA Wijnen^2^, Roelie M Wösten-van Asperen^1^, Marc R Lilien^5^, Marry M van den Heuvel-Eibrink^2,6^ , Marta F Fiocco^2,3,4^

**Index**

- **S1: Study design**
- **S2: Data collection**
- **S3: Definitions and measurements**
- **Figure S1: Study cohort**
- **Table S1: Nephrotoxic medication included in the study**
- **Table S2: Occurrence of AKI with highest stage of AKI during different episodes**
- **Table S3: Chronic Kidney Disease Outcomes Stratified by Duration of Acute**

**Kidney Injury**

- **Figure S2: Cumulative incidence per medicament with p-value from the Gray’s test**

**S1: Study design**

We conducted a retrospective cohort study of all pediatric cancer patients treated in the Princess Máxima Center between 1 January 2015 and 1 January 2021. The study started from the moment that treatment of all abdominal and thoracal tumors started to be nationally centralized. In that period regional pediatric hematological and neuro-oncological cancer patients were also treated in the Princess Máxima Center in Utrecht.

All patients who have given an informant consent to using their treatment data were included in this study. Data scientist of the Prinses Máxima Center has given all the data anonymous.

Demographic data were extracted from the electronic patient records. Treatment protocols were extracted from the National SKION® database (SKION)

**S2: Data collection**

Data were collected during the whole treatment period of each patient from the medical records. Baseline patient characteristics were obtained, including demographics (age, weight and gender), underlying cancer diagnosis, treatment protocols and the admissions of nephrotoxic medications.

Medication is classified as nephrotoxic if this was described as nephrotoxic in the pharmaceutic formulary of the Dutch hospitals, classified in 4 categories (>10%, 1-10%,0.1-1% or less 0.1%). We also classified medication as nephrotoxic if pharmacologist, pediatric oncologist, nephrologist or renal nurse it reviewed as nephrotoxic. More than 1200 medication were prescribed. Among these 1200 medications 51 were classified as nephrotoxic. Only medications who were administrated by minimal 50 patients were included in the multi state model.

The following clinical data were collected: initial blood test results, all available serum Cr levels, need of RRT, performance of nephrectomy as a part of the cancer treatment, relapse and HSCT data.

**S3: Definitions and Measurements**

Acute kidney injury was defined and classified according to the Kidney Disease: Improving Global Outcomes (KDIGO) criteria, which are based on changes of serum creatinine and urine output(1). Because of the retrospective nature of this study, and therefore not all the urine output were clearly noted, AKI was classified only at changes of serum Creatinine.


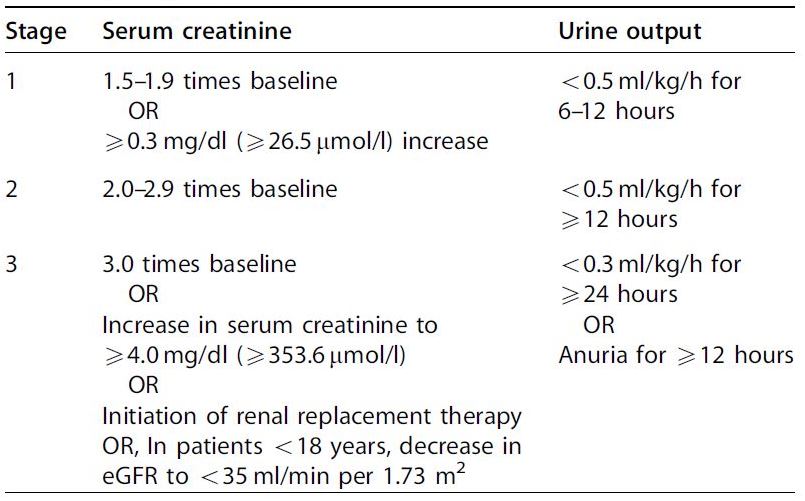


*AKI classified according KDIGIO criteria*

The baseline serum creatinine level was defined as the lowest serum level in the three months before hospital admission. When more serum creatinine levels were available, the baseline SCr was defined as the lowest SCr in the previous seven days. This strategy aimed to avoid underdiagnosis of AKI episodes in this population where it was anticipated that many children may develop low SCr levels due to loss of muscular cell mass, low protein intake, cachexia and inflammation(2). The eGFR was calculated using the modified Schwartz formula(3).

Data on maximum AKI stage, time from cancer diagnosis to AKI and number of AKI episodes were obtained.

After stop cancer treatment serum Creatinine levels were collected. CKD categories were classified according to 2012 KDIGIO classification (KDIGIO). Because of the lack in measurements of albuminuria due to the retrospective nature of the study, we classified CKD only based on eGFR. A final eGFR of less than 90 ml/minute/1.73m2 was defined as quick impaired renal function after stop therapy. The eGFR was calculated using the modified Schwartz formula.


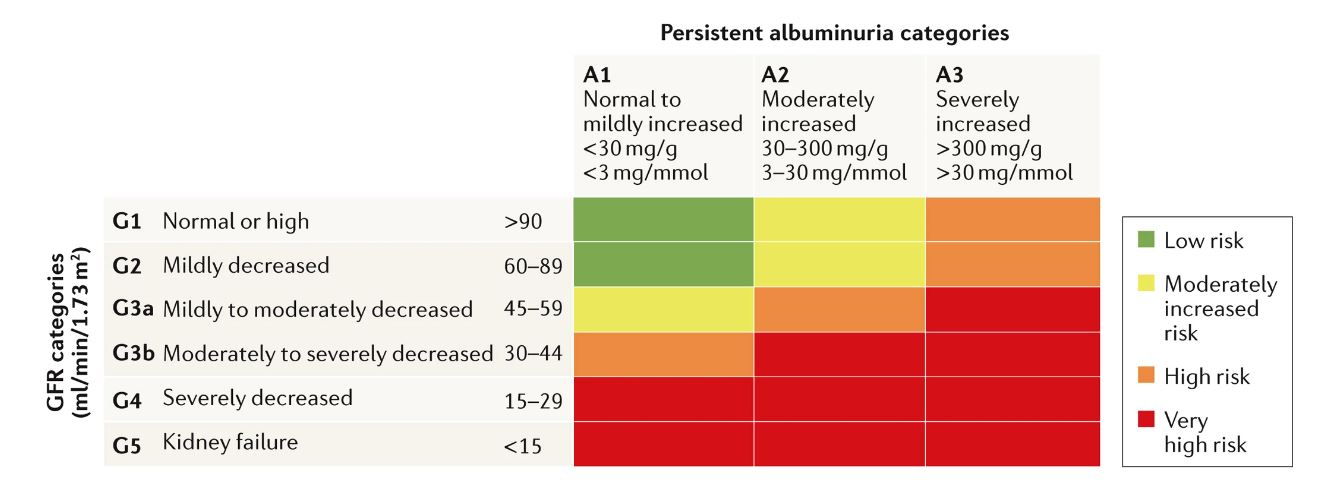


*CKD classified according KDIGIO criteria*

**Figure S1: Study cohort**

**Table S1. Nephrotoxic medication included in the study.**

| Medication | N | Total gifts | Medication | N | Total gifts |
| --- | --- | --- | --- | --- | --- |
| Acetylsalicylic acid (Aspirin) | 8 | 81 | Ibubrofen | 19 | 93 |
| acyclovir | 178 | 8697 | Ifosfamide | 343 | 6636 |
| Amphotericin B | 135 | 3914 | Indomethacin | 2 | 448 |
| Amikacin | 5 | 23 | Isavuconazole | 29 | 1446 |
| Co-trimoxazole | 590 | 6735 | lomustine | 28 | 129 |
| Biphosphanate | 18 | 60 | Methotrexaat | 516 | 17655 |
| Bleomycin | 8 | 45 | Micafungin | 331 | 3720 |
| Busulfan | 96 | 800 | Morphine | 477 | 4756 |
| Carboplatin | 184 | 1643 | Oxaliplatin | 2 | 10 |
| Caspofungin | 8 | 47 | Pentamidine | 146 | 946 |
| Ceftazidime | 629 | 17073 | Piperacillin-tazobactam | 41 | 299 |
| Cefuroxime | 9 | 75 | Posaconazole | 36 | 923 |
| Celecoxib | 69 | 1093 | Rifampin | 10 | 256 |
| Ciclosporin | 61 | 2340 | Rituximab | 51 | 280 |
| Cidofovir | 3 | 29 | Sirolimus | 7 | 70 |
| Cisplatin | 295 | 2885 | Spironolactone | 171 | 4770 |
| Clindamycin | 66 | 899 | Tacrolimus | 19 | 667 |
| Cyclophosphamide | 723 | 12582 | Teicoplanin | 185 | 1187 |
| Diclofenac | 330 | 2053 | Tobramycin | 7 | 67 |
| Enalapril | 109 | 2873 | Valacyclovir | 193 | 4644 |
| Etoposide | 567 | 7763 | Valganciclovir | 14 | 88 |
| Evoltra/ Clofarabine | 9 | 72 | Vancomycin | 521 | 26564 |
| Fluconazole | 97 | 1057 | oriconazole | 117 | 6366 |
| Foscarnet | 10 | 310 |  |  |  |
| Furosemide | 401 | 12246 |  |  |  |
| Ganciclovir | 5 | 104 |  |  |  |
| Gentamicin | 177 | 513 |  |  |  |
| Hydrochloortiazide | 35 | 1037 |  |  |  |

*Only medications n>10 AKI events are included in the cause specific hazard model*

**Table S2:** **Severity and duration of different AKI episodes.**

| AKI severity  (KDIGO) | AKI Episode 1  N=570 | AKI Episode 2  N=234 | AKI Episode >=3  N=97 |
| --- | --- | --- | --- |
| Stage 1 | 371 (65.1) | 129 (55.1) | 28 (28.9) |
| Stage 2 | 135 (23.7) | 65 (27.8) | 42 (43.3) |
| Stage 3 | 64 (11.2) | 40 (17.1) | 26 (26.8) |

| Duration of AKI Episode | AKI Episode 1  N=570 | AKI episode 2  N=234 | AKI episode >=3  N=97 |
| --- | --- | --- | --- |
| Transient AKI (<2days) | 324 (56.8) | 92 (39.3) | 14 (14.4) |
| Persistent AKI (2-7 days) | 131 (23.0) | 67 (28.6) | 26 (26.8) |
| Acute Kidney Disease (>7days) | 115 (20.2) | 75 (32.1) | 57 (58.8) |

**Table S3: Chronic Kidney Disease Outcomes Stratified by Duration of Acute Kidney Injury.**

|  | **No CKD**  **N=968** | | | **CKD**  **N=191** | | |
| --- | --- | --- | --- | --- | --- | --- |
|  | **1 episode of AKI**  **N=217** | **2 episodes of AKI**  **N= 75** | **>3 episodes of AKI**  **N= 31** | **1 episode of AKI**  **N=42** | **2 episodes of AKI**  **N=36** | **>3 episodes of AKI**  **N=61** |
| Transient AKI  (< 2 days) n (%) | 147/217 (68) | 31/75 (41) | 3/31 (10) | 10/42 (24) | 7/36 (19) | 3/61 (5) |
| Persistent AKI  ( 2-7 days) n (%) | 49/217 (22) | 28/75 (37) | 15/31 (48) | 4/42 (10) | 9/36 (25) | 7/61 (11) |
| Acute Kidney Disease  ( > 7 days) n (%) | 21/217 (10) | 16/75 (21) | 13/31 (42) | 28/42 (67) | 24/36 (67) | 55/61 (90) |
| No AKI | 645 | | | 44 | | |

**Figure S2: Cumulative incidence of AKI for each medicament.**


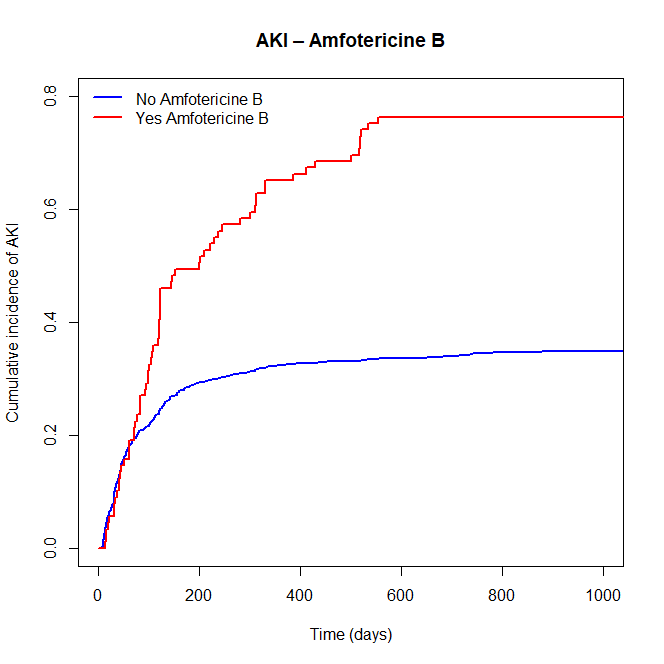

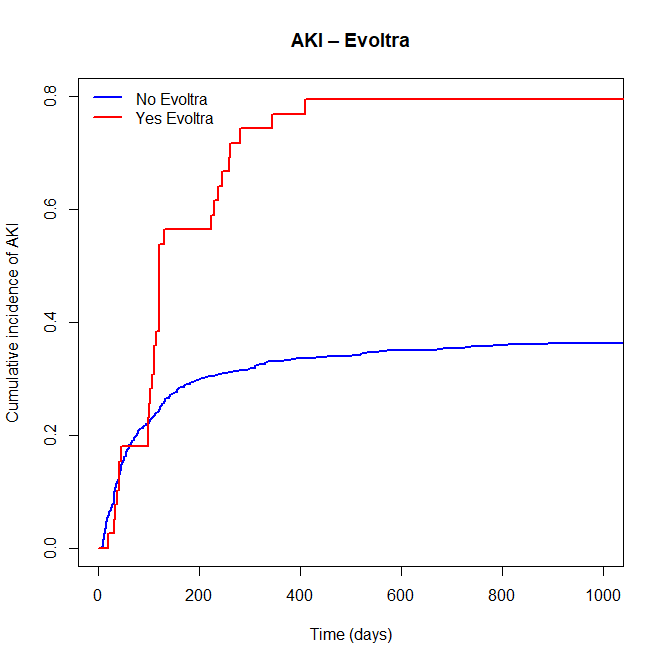

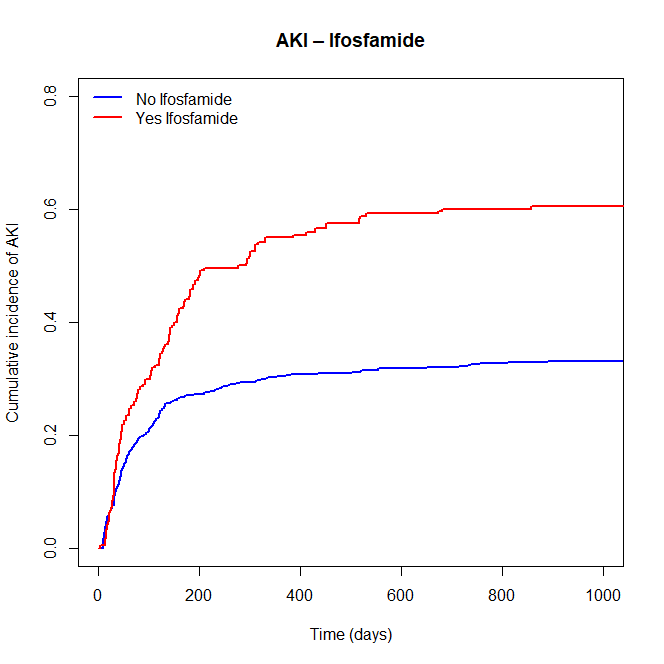

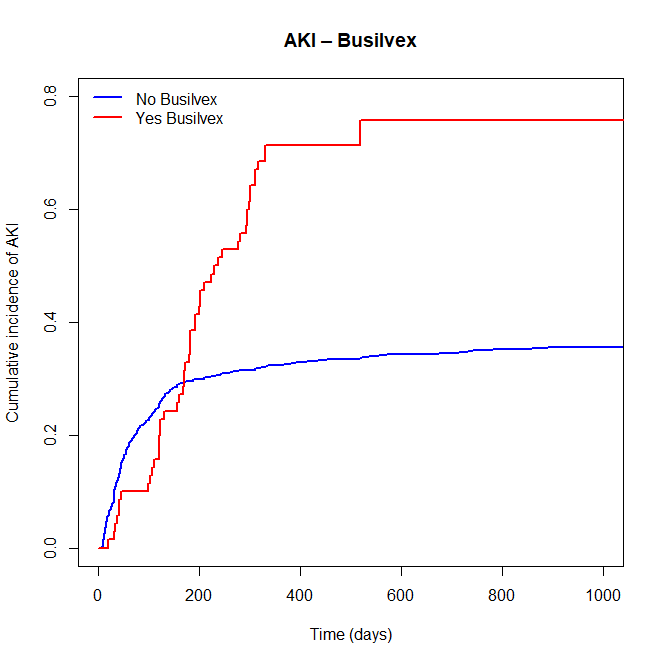


Gray’s test: p <0.001

Gray’s test: p <0.001

Gray’s test: p <0.001

Gray’s test: p <0.001


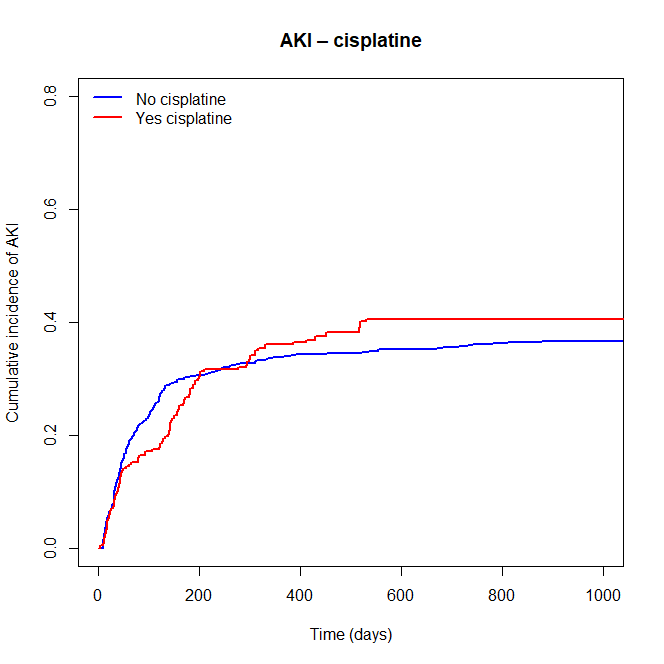

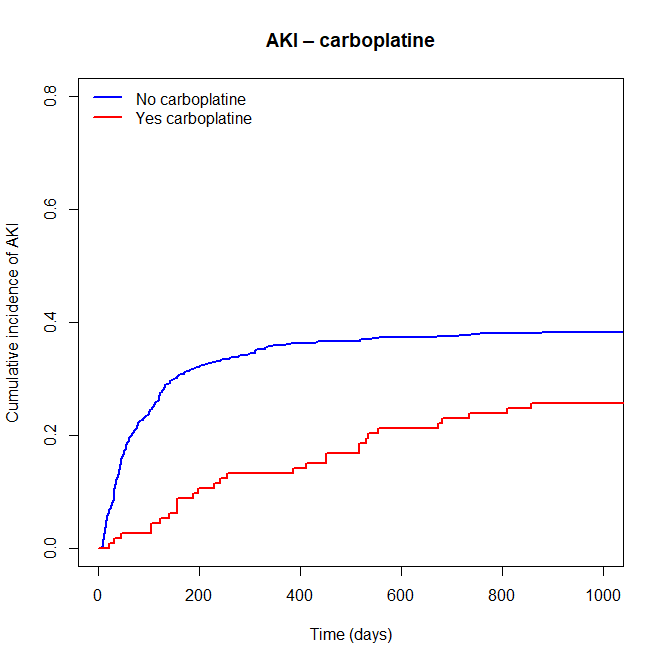


Gray’s test: p = 0.491

Gray’s test: p = 0.001


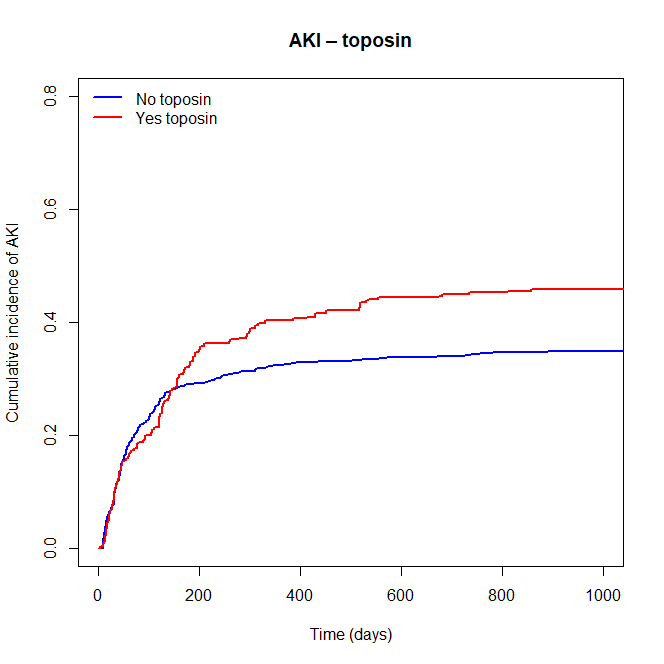

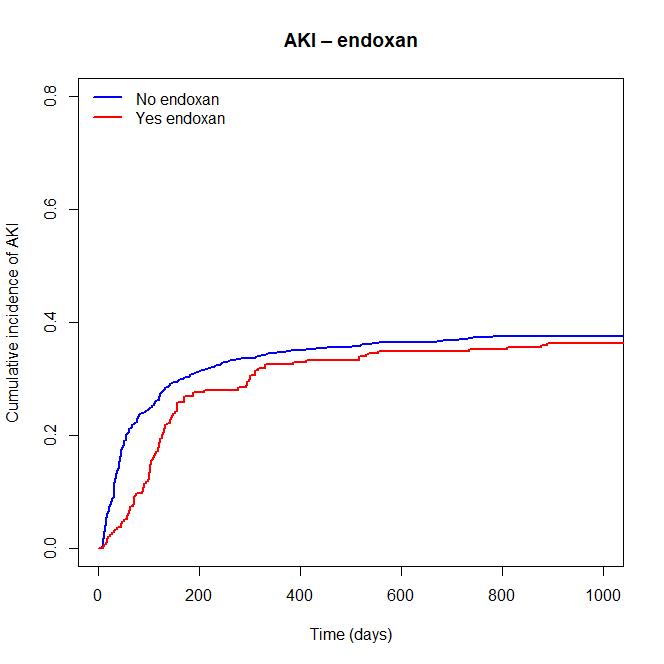

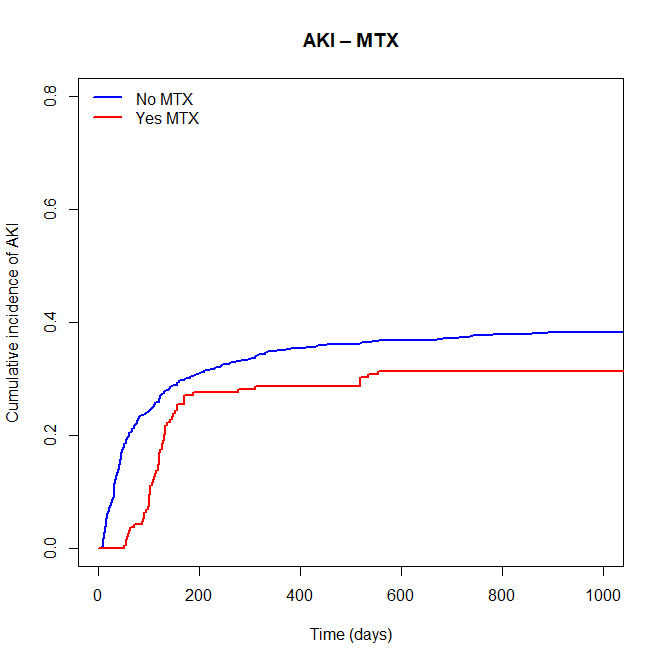


Gray’s test: p = 0.021

Gray’s test: p = 0.240

Gray’s test: p = 0.002


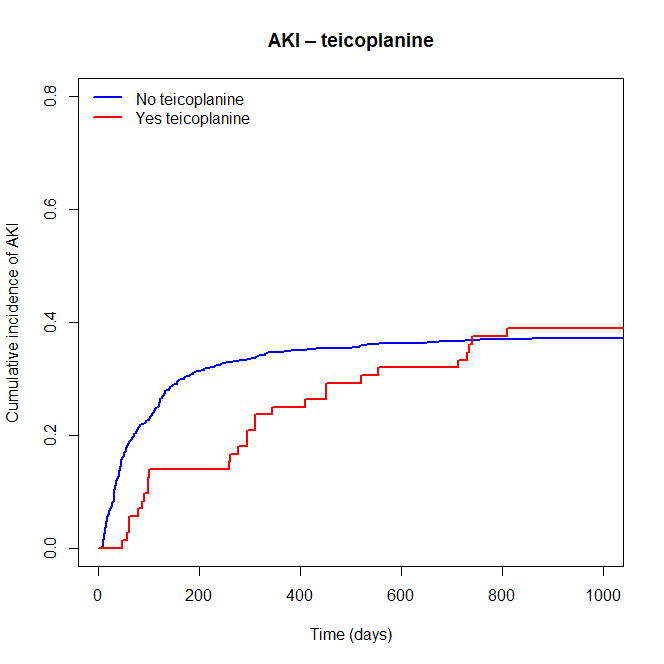

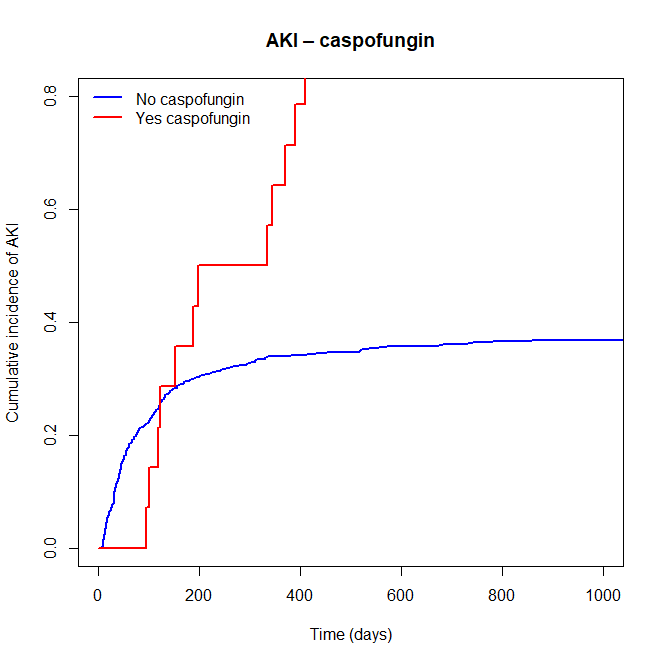

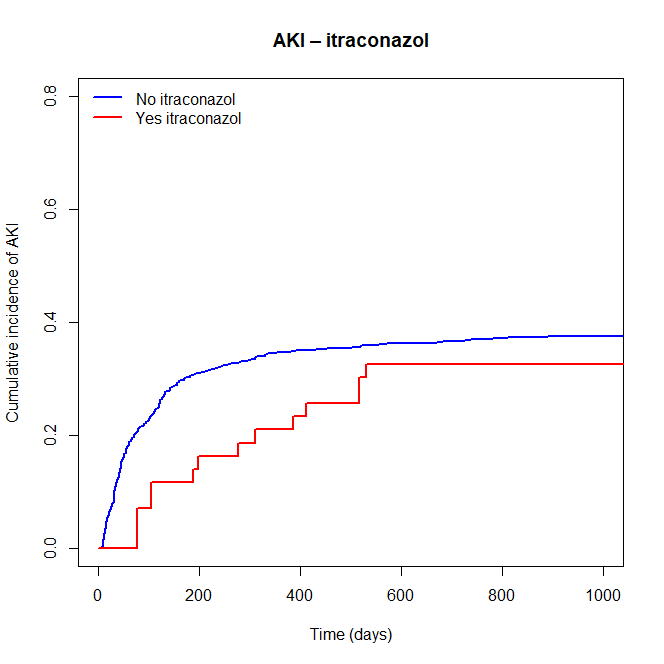

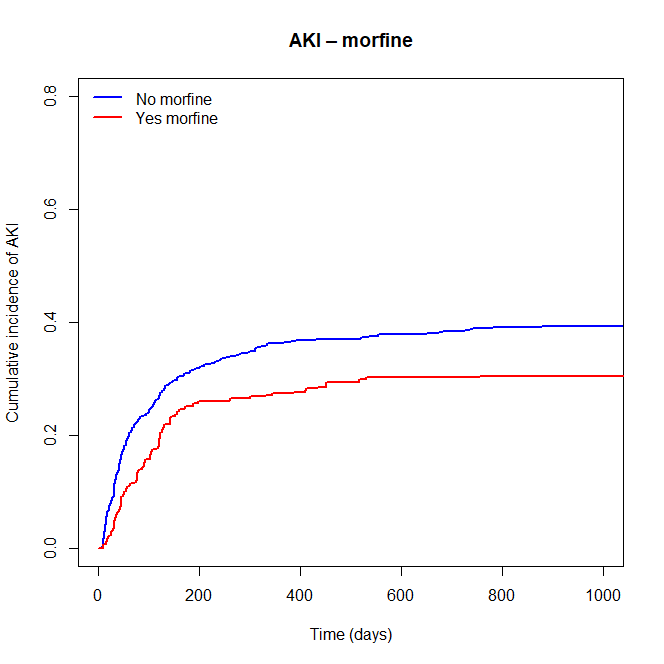


Gray’s test: p = 0.645

Gray’s test: p = 0.003

Gray’s test: p = 0.012

Gray’s test: p = 0.278


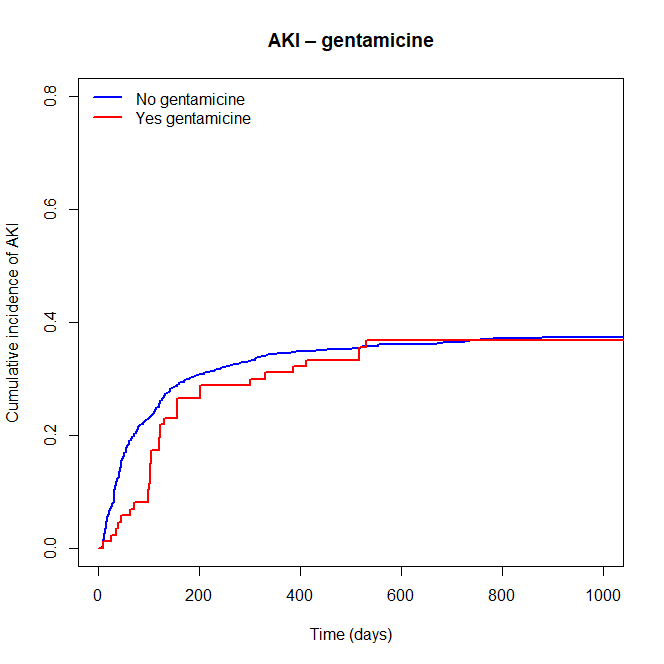

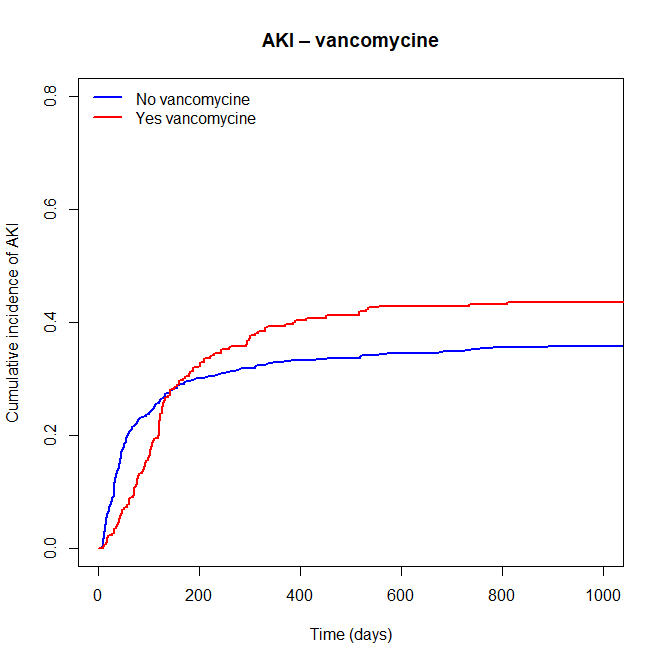

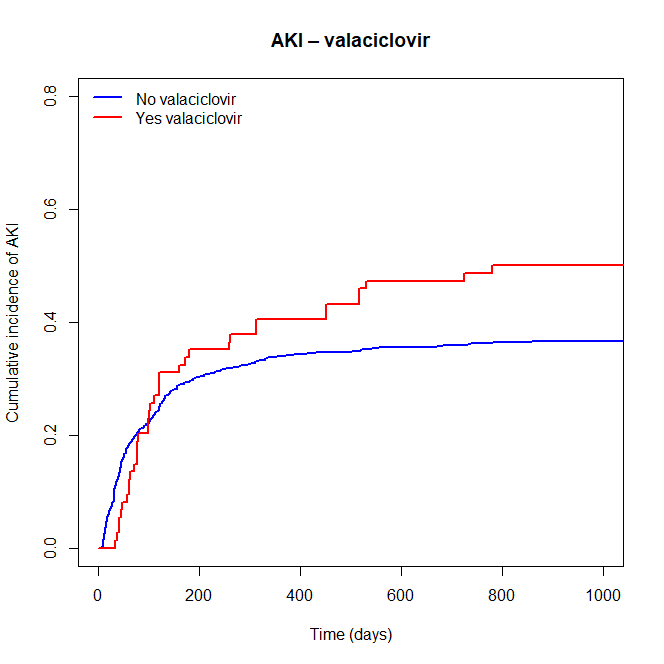

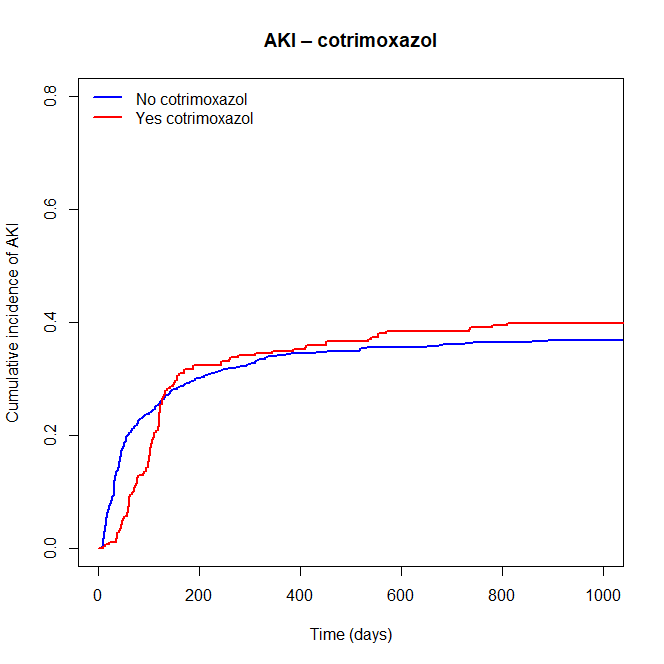

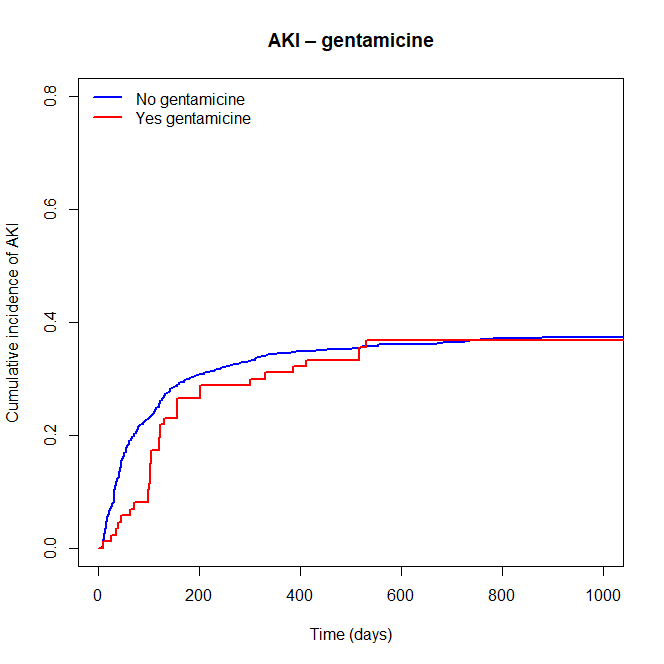

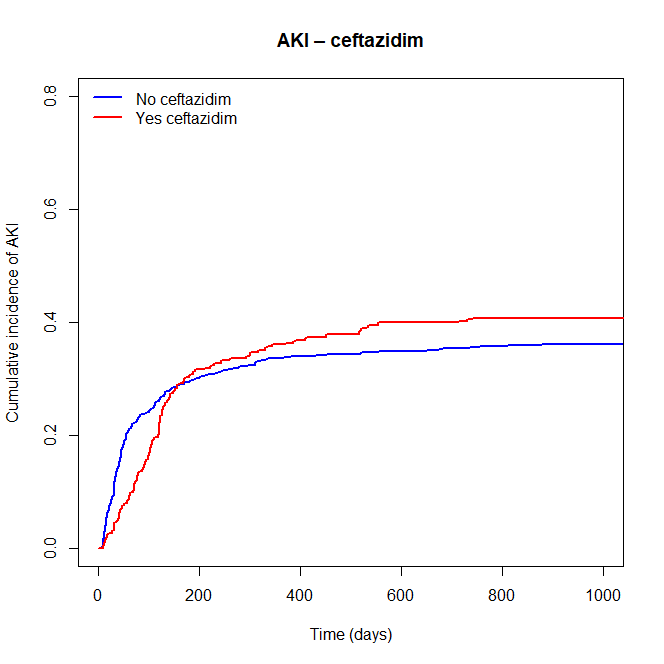


Gray’s test: p = 0.847

Gray’s test: p = 0.111

Gray’s test: p = 0.619

Gray’s test: p = 0.076

Gray’s test: p = 0.619

Gray’s test: p = 0.456

**Reference**

1. KDIGO clinical guideline. 2012.

2. Lameire NH, Flombaum CD, Moreau D, Ronco C. Acute renal failure in cancer patients. Annals of Medicine. 2005;37(1):13-25.

3. Schwartz GJ, Munoz A, Schneider MF, Mak RH, Kaskel F, Warady BA, Furth SL. New equations to estimate GFR in children with CKD. Journal of the American Society of Nephrology : JASN. 2009;20(3):629-37.

4. Gray R.J. A Class of K -Sample Tests for Comparing the Cumulative Incidence of a Competing Risk. The annals of statistics. 1988;16(3):1141-54.
